# Answers to Querists

**Published:** 1867-11

**Authors:** 


ANSWERS TO QUERISTS.

*Properties, Generation and Administration of Nitrous Oxide Gas.* Query 2nd.—*Generation of Nitrous Oxide Gas? Answer Continued.*

THE gasometer being in position, and connected by means of rubber tubing with the wash-bottles, the stopcock turned on, and an extra weight added to the cords passing over the pulleys, to assist the ascent of the bell or receiver as the gas enters it, we proceed as follows:

Fill the retort half or two-thirds full, according to the quantity of gas desired. A medium sized retort filled half full of the crystalized nitrate of ammonia will generate about thirty gallons of the gas, after the water in the gasometer has become impregnated with it. The crystalized nitrate of ammonia is preferable to the fused ammonia for the reason that pure white crystals cannot be made from an impure article of this salt; while the fused preparation may contain certain impurities without our being able so readily to detect them.

When, however, we have reason to suspect the impurity of the salt in use, such for example, as the presence of the chloride of ammonium (nitrate of ammonia,) which would liberate chlorine when it is decomposed by heat, it may be tested for this impurity as follows: "dissolve some of the nitrate of ammonia in pure water; also dissolve a a small quantity of the nitrate of silver in pure water, and pour the solution of the nitrate of ammonia gradually into that of the nitrate of silver; if any soluble chloride is present, a grayish white precipitate will first be formed, which will render the solution milky in appearance, the precipitate gradually settling to the bottom."

The retort containing the ammonia is then connected by means of rubber tubing with wash-bottle No. 1, and heat applied by means of a gas, kerosene, or alcohol lamp.

Instead of slipping one end of the rubber tubing over the neck of the retort and thus making the connection with the wash-bottles, as is frequently done, a soft rubber stopper should fit closely the orifice in the neck, with a short glass tube passing through this stopper; the rubber tubing can then be slipped over the projecting end of this glass tube. By making such a connection as this, the rubber tubing is preserved in a great measure from the heat of the retort, which otherwise soon destroys it.

Where gas is used under the retort it should burn as low as possible until the ammonia begins to melt; then more heat can be applied by increasing the flame, and the liquid ammonia be brought to the boiling point.

The same rule is applicable where kerosene and alcohol are used to generate the gas. Care is necessary, both for the purity of the gas and the safety of the retort, that the ebulition be not too rapid, *as white fumes should never ap-*

*pear in the wash–bottles.* Should the heat be too intense the ammonia "will be volatalized and wasted, as will be indicated by the the appearance of a white cloud instead of a colorless gas, or its decomposition will be attended by the formation of such objectionable compounds as nitric oxide and hyponitrous acid, either of which will, of course, necessitate the reduction of heat and careful purification; other impurities may also be given off, such as nitric acid and chlorine." Where the ordinary gas, kerosene or alcohol lamp is used under the retort, the heat may be regulated by increasing or diminishing the flame.

Sprague's apparatus has attached to it an automatic regulator, by which a uniform temperature of the ammonia can be maintained during the entire process of generating the gas.

The lamp used in generating the gas, should be removed before all the ammonia in the retort is exhausted; otherwise the retort is liable to be fractured by the heat.

After the removal of the heat from the retort, and as soon as the gas ceases to bubble up through the liquid in the first wash-bottle, the connection with the retort should be severed by removing the rubber tubing from where it is connected at the orifice of the neck.

Neglect in doing this will result in the fracture of the retort, from a vacuum being formed by the condensation of the steam, the atmospheric pressure drawing the contents of the first wash-bottle over into it.

The desired quantity of gas being obtained, the small stop-cock at the base of the gasometer, which allows the gas from the wash-bottles or purifier to enter the receiver, should be closed; the extra weights, before referred to, should be removed as the remaining ones balance the bell in the tank.

It is also advisable to remove all except the upper and smaller weights when the patient is inhaling the gas, that no obstruction may be offered to its flow from the receiver into the inhaling tube.

